# The potential for utilising in‐hospital glucose measurements to detect individuals at high risk of previously undiagnosed diabetes: Retrospective cohort study

**DOI:** 10.1111/dme.14918

**Published:** 2022-07-26

**Authors:** Andrew J. Farmer, Brian Shine, Laura C. Armitage, Noel Murphy, Tim James, Nishan Guha, Rustam Rea

**Affiliations:** ^1^ Nuffield Department of Primary Care Health Sciences University of Oxford Oxford UK; ^2^ Oxford University Hospitals NHS Foundation Trust Oxford UK

**Keywords:** cohort study, diabetes, in‐hospital testing, diagnosis, digital health

## Abstract

**Background:**

Many people with undiagnosed diabetes have hyperglycaemia when admitted to hospital. Inpatient hyperglycaemia can be an indication of diabetes mellitus but can also indicate a stress response. This study reports the extent to which an in‐hospital maximum observed random glucose measurement is an indicator of the need for in‐hospital (or subsequent) HbA1c measurement to look for undiagnosed diabetes.

**Methods:**

Blood glucose, HbA1c, age and sex were collected for all adults following admission to a UK NHS trust hospital from 1 January 2019 to 31 December 2020. We restricted the analysis to those participants who were registered with a GP practice that uses the trust laboratory and who had at least some tests requested by those practices since 2008. We stratified individuals according to their maximum in‐hospital glucose measurement and report the number of these with HbA1c measurement ≥48 mmol/mol (6.5%) prior to the index admission, and during and after admission. We calculated an estimated proportion of individuals in each blood glucose stratum without a follow‐up HbA1c who could have undiagnosed diabetes.

**Results:**

In toal, 764,241 glucose measurements were recorded for 81,763 individuals who were admitted to the Oxford University Hospitals Trust. The median (Q1, Q3) age was 70 (56, 81) years, and 53% were males. Of the population, 70.7% of individuals declared themselves to be of White ethnicity, 3.1% of Asian background, and 1.1% of Black background, with 23.1% unstated. Of those individuals, 22,375 (27.4%) had no previous HbA1c measurement recorded. A total of 1689 individuals had a diabetes‐range HbA1c during or after their hospital admission (2.5%) while we estimate an additional 1496 (2.2%) may have undiagnosed diabetes, with the greatest proportion of these having an in‐hospital glucose of ≥15 mmol/L. We estimate that the number needed to detect a possible new case of diabetes falls from 16 (in‐hospital glucose 8 mmol/L to <9 mmol/L) to 4 (14 mmol/L to <15 mmol/L).

**Conclusion:**

The number of people who need to be tested to identify an individual who may have diabetes decreases as a testing threshold based on maximum in‐hospital glucose concentration increases. Among those with hyperglycaemia and no previous HbA1c measurement in the diabetes range, there appears to be a lack of subsequent HbA1c measurement. This work identifies the potential for integrating the testing and follow‐up of people, with apparently unrecognised hospital hyperglycaemia across primary and secondary care.

**Novelty statement**

- This analysis of hospital laboratory audit data has identified the potential for identifying people with a diabetes‐range HbA1c during or after an elevated in‐hospital glucose measurement.
- Just over half of those with elevated in‐hospital glucose measurements and no available pre‐admission HbA1c measurement in the diabetes‐range had a subsequent HbA1c measurement.
- Routinely measuring HbA1c at higher maximum blood glucose strata can identify people with a HbA1c in the diabetes range and could be an efficient means to detect people with undiagnosed diabetes.

## 1 INTRODUCTION

Many people with undiagnosed diabetes have hyperglycaemia when they are admitted to hospital.^1^, ^2^, ^3^ There is no accepted systematic process of diagnosing diabetes within this population although there is evidence that undiagnosed diabetes is more prevalent in hospital inpatients than the general population.^4^

Inpatient hyperglycaemia can be an indication of diabetes mellitus but can also indicate a stress response. Determining the cause of hyperglycaemia in people presenting to hospital is thus a challenge. A large cohort study showed that elevated random glucose levels on admission to hospital are associated with increased risk of both a subsequent diagnosis of diabetes over a 3‐year period, and death.^5^ In this study, 77% of people admitted with blood sugars of greater than 11.1 mmol/L did not have a diagnosis of diabetes at 3 years.^5^ This poses the question of what threshold of hyperglycaemia should prompt further investigation to establish a diagnosis of diabetes.

This study aims to explore the potential for elevated in‐hospital glucose measurements as a trigger for HbA1c measurement. Similar studies have been carried out with a wide range of parameters for testing. For example, one study examined the use of automatically triggered HbA1c measurements for 133,837 people admitted to secondary and tertiary hospitals in New South Wales, Australia, during 2011 and 2012. The study used a plasma glucose value of >14 mmol/L as the cut‐off.^6^ The authors cite “stress hyperglycaemia” as the main differential diagnosis in the inpatient with hyperglycaemia. The rationale for the decision to use >14 mmol/L as a cut‐off in this study was the risk of overburdening the clinicians if a lower value was used.^2^ The authors report that automatically triggering HbA1c measurements in their intervention group did not lead to increased rates of diabetes diagnoses in hospital.^6^ Another Australian study used a cut‐off of 5.5 mmol/L among people presenting to the emergency department to trigger HbA1c measurement.^3^ They found that 11% of those tested had HbA1c measurements consistent with diabetes.^7^ A more diagnostically relevant threshold of 11.1 mmol/L may increase the accuracy of detection, but this has not been confirmed.

A recent systematic review of this field concluded that, although several studies have investigated the rate of undiagnosed diabetes among individuals with an in‐hospital glucose measurement above a defined threshold, there is a lack of diagnostic accuracy data and a lack of consensus on the most appropriate glucose threshold above which formal diagnostic testing should be performed.^8^, ^9^ Recent guidance suggests measurement of HbA1c where glucose measurement is 7.8 mmol/L in individuals without a known diagnosis of diabetes, but this recommendation is based on expert opinion and the evidence for the threshold proposed is unclear.^10^

We have examined the relationship between routinely collected in‐hospital glucose measurements and the level of subsequently measured HbA1c to explore whether plasma (adjusted) glucose‐guided HbA1c measurement might be an efficient way of testing for diabetes in adults admitted to hospital and establish the potential for translating this approach into clinical practice.

## 2 METHODS

This study was carried out using data from adults aged 18 years or older admitted to the Oxford University Hospitals NHS Foundation Trust (OUH) from 1 January 2019 to 31 December 2020. The data held in the hospital laboratory database were used for this analysis. The data used for this analysis were not linked to other sources of information in the hospital electronic record. We restricted this analysis to participants registered with a GP practice that sends requests to our laboratory, and for whom at least one request from primary care had been received since the beginning of 2008.

Anonymised laboratory data were extracted for all individuals with a glucose measurement. Methods for glucose measurement used within the hospital depend on the clinical setting. Comparability of measurement across the different devices and assays is supported by internal quality control and participation in external quality assurance programmes. Methods used were the point of care, handheld Abbott Precision Pro (Abbott Diabetes Care UK), the desktop Radiometer blood gas range (Radiometer UK Ltd.), and the portable Abbott i‐stat (Abbott Rapid Diagnostics Ltd) analyser. Within the laboratory the Abbott c16000 chemistry analysers (Abbott Laboratories Ltd) was used. Where required instruments were set to report plasma glucose equivalents. We have referred to plasma glucose measurements throughout the manuscript to include adjusted plasma glucose measurements. The hospital data did not include information about the fasting status of participants alongside the glucose measurement.

HbA1c measurements included those requested from hospital and from general practices in the catchment area of the hospital (Oxfordshire, and areas overlapping with Berkshire, Northamptonshire, and Buckinghamshire). The hospital laboratory is the sole laboratory used for NHS laboratory work in this area. All HbA1c testing used ion‐exchange chromatography analysis with the Bio‐Rad D100(Bio‐Rad Clinical Diagnostics, Watford, UK). HbA1c measurements were available from 1/1/2008 to 30/11/2021.

We extracted the age and sex of individuals, ethnicity, practice identifier for the participant, date of admission and the date and time and results of all glucose and HbA1c measurements.

We included all individuals with an in‐hospital recorded plasma glucose ≥4 mmol/L during and following their first admission during the period of this analysis. We stratified the data for each individual by their maximum glucose concentration during the hospital admission. Data are presented on the number of people with

- a maximum random glucose measurement presented in 1 mmol/L strata, with a cut point below 5 mmol/L and above 15 mmol/L where ketone testing is recommended,
- a prior HbA1c measurement ≥48 mmol/mol (6.5%) (representing a diabetes‐range HbA1c measurement and a surrogate for a prior diabetes diagnosis);
- HbA1c testing during and after admission defined as after the date of the maximum glucose measurement identified for the purposes of this analysis,
- without a previous HbA1c measurement but with an in‐hospital or follow‐up HbA1c measurement and
- a HbA1c ≥48 mmol/mol (6.5%) measured during or after admission but no pre‐admission diabetes range HbA1c measurement (considered to represent people with potentially undiagnosed diabetes).

These data were used to calculate the expected proportion of individuals in each stratum without an available post‐admission HbA1c measurement who were likely to have an HbA1c ≥48 mmol/mol (6.5%) (a surrogate marker for unrecognised diabetes). We assumed, for the purposes of the analysis, that the probability of a diabetes‐range HbA1c was similar for those with in and post‐hospital HbA1c testing, and for those who were not tested. Data on individuals readmitted with subsequent in‐hospital glucose measurements were included with data from the first admission.

### 2.1 Statistical analysis

All data from analysers were automatically uploaded to the laboratory database and then extracted as an anonymised database onto secure Trust servers for analysis. Data were analysed using the statistical package R.^11^ Counts and percentages are presented by category. Median, 25th (Q1) and 75th (Q2) centiles are presented where appropriate. Number needed to detect were calculated as the number of people that would be tested divided by the number of individuals detected as having a diabetes level HbA1c measurement, with the number approaching 1 for a completely efficient test.

This project was completed as an approved local audit at the Oxford University Hospitals NHS Foundation Trust (May 2021), approval number 6928.

## 3 RESULTS

Between 1 January 2019 and 31 December 2020, 764,241 glucose measurements were recorded for 81,763 individuals admitted to the Oxford University Hospitals Trust registered with a practice using the hospital laboratory. The median (Q1, Q3) age was 70 (56, 81) years, and 53% were males. Of the population, 70.7% of individuals declared themselves to be of White ethnicity, 3.1% of Asian background and 1.1% of Black background, with 23.1% unstated. The median number of admissions was 2,^1^, ^3^ and median length of admission was 1.3 (0.3, 3.5) days.

Of the glucose measurements, 459,940 (60.2%) were made using a point‐of‐care analyser, 265,233 (34.7%) on blood gas instruments, 25,782 (3.4%) in the laboratory, and 13,286 (1.7%) on a portable analyser. 143,265 (18.7%) of the glucose measurements were made on the day of the first admission, 51,995 (4.0%) on days 1 and 2, 30,036 (3.9%) on days 3 to 5, and 538,945 (70.5%) on day 5 of the admission or a subsequent admission.

**FIGURE 1:**
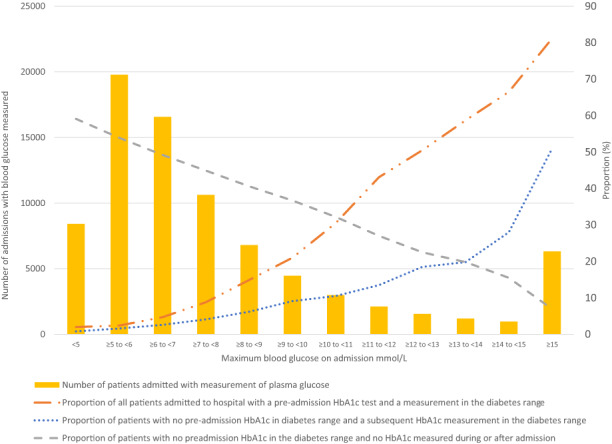
Number of patients admitted with measurement of glucose; and proportions of patients with pre‐admission HbA1c in the diabetes range, occurrence of diabetes range HbA1c measurements during and after admission, and HbA1c not measured during or after admission (*n* = 81,763).

Results are presented by random glucose stratum for the maximum glucose observed (Figure 1). The number of participants included in each maximum‐glucose stratum decreases as the glucose level increases. There were 22,375 (27.4%) individuals with no previous measurement of HbA1c (Table 1), decreasing to 12.0% for those with a glucose ≥9 mmol/L (Table 1). 16.4% of all participants (13,396 individuals) had a HbA1c value in the diabetes range prior to the first admission, rising to 81.2% (5124 of 6312) with a maximum glucose ≥15 mmol/L (Table 1).

**TABLE 1:** Measurement of HbA1c during or after‐hospital admission over 2‐years by maximum glucose (mmol/L) (*n* = 81,763)

Glucose Stratum (mmol/L)	Numbers admitted to hospital with in‐hospital maximum glucose measurement	HbA1c measured before hospital admission (*n*)	Proportion with HbA1c measured before hospital admission (%)	Diabetes‐range HbA1c recorded before hospital admission (*n*)	Proportion with diabetes‐range HbA1c recorded before hospital admission (%)	No prior diabetes range HbA1c measurement before hospital admission (*n*,%)	Individuals with HbA1c measurement during or after hospital admission (*n*)	Proportion with HbA1c measurement during or after hospital admission (%)	First HbA1c measurement during or after admission (n,%)
<5	8416	4708	55.9	171	2.0	8245 (98.0%)	3423	40.7	1064 (12.6%)
≥5 to <6	19,774	12,607	63.8	477	2.4	19,297 (97.6%)	9083	45.9	2309 (11.7%)
≥6 to <7	16,560	11,566	69.8	784	4.7	15,776 (95.3%)	8338	50.4	1718 (10.4%)
≥7 to <8	10,616	7918	74.6	934	8.8	9682 (91.2%)	5768	54.3	976 (9.2%)
≥8 to <9	6803	5346	78.6	1012	14.9	5791 (85.1%)	3935	57.8	551 (8.1%)
≥9 to <10	4458	3612	81.0	938	21.0	3520 (79.0%)	2720	61.0	350 (7.9%)
≥10 to <11	2993	2503	83.6	914	30.5	2079 (69.5%)	1931	64.5	206 (6.9%)
≥11 to <12	2109	1821	86.3	909	43.1	1200 (56.9%)	1446	68.6	128 (6.1%)
≥12 to <13	1559	1380	88.5	788	50.5	771 (49.5%	1129	72.4	85 (5.5%)
≥13 to <14	1194	1093	91.5	701	58.7	493 (41.3%)	881	73.8	54 (4.5%)
≥14 to <15	969	892	92.1	644	66.5	325 (33.5%)	750	77.4	37 (3.8%)
≥15	6312	5942	94.1	5124	81.2	1188 (18.8%)	5347	84.7	255 (4.0%)
*N* (columns)	81,763	59,388		13,396		68,367 (83.6%)	44,751		7733 (9.5%)

Of those participants with a pre‐admission HbA1c measurement available, the proportion of those with a diabetes‐range HbA1c increased by stratum of glucose (Table 1 and Figure 1). Of those participants with no HbA1c in the diabetes range prior to admission, and with a HbA1c measurement during or after admission, 1689 (2.5%) individuals were identified with a diabetes‐range HbA1c., while we estimate an additional 1496 (2.2%) may have had a diabetes‐range HbA1c if they had been tested (Table 2).

**TABLE 2:** Detection of diabetes level HbA1c during or after admission over 2‐years by in‐hospital maximum glucose (mmol/L) measured

Glucose stratum (mmol/L)	Number of patients without prior diabetes‐range HbA1c measurement and with first HbA1c ≥48mmol/mol measured during or after hospital admission	Proportion of total number admitted to hospital with first HbA1c ≥48mmol/mol) detected during or after admission (%)	Proportion with first HbA1c ≥48mmol/mol detected during or after hospital admission as a proportion of those without prior diabetes range HbA1c measurement (%)	No of patients with no measurement of HbA1c during or after admission and no prior diabetes range HbA1c measured	Proportion of all patients with no measurement of HbA1c during or after admission and no prior diabetes range HbA1c (%)	Proportion of those not known to have diabetes with HbA1c not measured (%)	Potential number of people with undetected HbA1c in diabetes range	Total numbers of people that could be detected with systematic testing	Number needed to detect
<5	27	0.3	0.8	4972	59.1	60.3	41	68	121
≥5 to <6	142	0.7	1.6	10651	53.9	55.2	175	317	61
≥6 to <7	195	1.2	2.6	8153	49.2	51.7	209	404	39
≥7 to <8	201	1.9	4.1	4766	44.9	49.2	195	396	24
≥8 to <9	189	2.8	6.2	2765	40.6	47.7	173	362	16
≥9 to <10	172	3.9	9.1	1638	36.7	46.5	150	322	11
≥10 to <11	117	3.9	10.5	964	32.2	46.4	101	218	10
≥11 to <12	85	4.0	13.5	570	27.0	47.5	77	162	7
≥12 to <13	78	5.0	18.5	350	22.5	45.4	65	143	5
≥13 to <14	51	4.3	19.8	236	19.8	47.9	47	98	5
≥14 to <15	49	5.1	28.0	150	15.5	46.2	42	91	4
≥15	383	6.1	51.0	437	6.9	36.8	223	606	2
Column (*n*)	1689			35652			1498	3187	

For 87.7% of specimens, a suitable ethylenediamine tetra acetic acid (EDTA) specimen for measuring HbA1c was available taken within the previous 72 h or in the following 12 h.

## 4 DISCUSSION

### 4.1 Findings

This work confirms that many patients admitted to hospital may not have a HbA1c measurement following an elevated in‐hospital glucose measurement. Among the cohorts with high glucose levels, between 45% and 48% of those individuals not known to have a prior diabetes‐range HbA1c were identified as not having a subsequent HbA1c measurement. Among those that were tested during or after admission, the proportion of participants with a diabetes range HbA1c appears to be sufficient for substantial numbers of people to be identified with diabetes, with the numbers needed to test to identify a case of diabetes falling as maximum glucose concentration increases (Table 2). Corresponding estimates of the additional workload this strategy would involve are also presented (Table 3).

**TABLE 3:** Numbers of patients with maximum glucose above threshold and projected number of HbA1c tests

Random glucose threshold (mmol/L)	Population above the glucose threshold over 2‐year period	Number of HbA1cs that would be performed^a^ in each week if the corresponding random glucose threshold was applied
≥7	41,034	395
≥8	29,304	281
≥9	21,831	107
≥10	16,862	82
≥11	13,363	65
≥12	10,991	53
≥13	9181	45
≥14	7821	38
≥15	6733	33

^a^ In the Oxford University Hospitals NHS Trust.

The proportion of participants identified with a diabetes range HbA1c during or after admission appeared to increase among those with a glucose of 9 mmol/L and above during their hospital admission. This work provides preliminary information about the potential value of targeted testing at different glucose thresholds.

### 4.2 Comparison with the literature

This is a detailed retrospective audit, reporting data from a notably larger cohort than those studies previously undertaken. A systematic review of previous studies using in‐hospital glucose measurements to detect diabetes^8^ identified 12 relevant studies, but mostly with small numbers of screened participants, the largest of which had a study population of 16,268 individuals.^12^

Population screening strategies for diabetes have been previously evaluated and comparison with these studies can be more informative than comparison of numbers needing to test (NNT) for other conditions where the invasiveness, cost and outcomes of testing may be different. A population study in Ontario indicated an NNT to identify undiagnosed diabetes was 14 among men and 22 among women.^13^ A study of testing in health records from general practice based on age and BMI suggests a range of NNT 7 to 12.^14^ The data provided in our study suggest that focussing on those with higher levels of glucose could offer a more efficient strategy for predicting the occurrence of diabetes‐level HbA1c among those admitted to hospital.

This work informs future potential practice where data could be automatically captured not only from laboratory measurements but also point‐of‐care measurements with glucometers and blood gas analysers. It adds to a detailed audit previously carried out describing measurement of glucose levels for acute admissions and the characteristics of those measured and not measured noting “…there is little evidence on how many cases of diabetes would be confirmed on HbA1c testing.”^9^ This analysis starts to provide that information.

### 4.3 Strengths and Limitations of this analysis

This work includes an analysis of more than 80,000 individuals and integrates primary and secondary care HbA1c data to allow accurate characterisation of participants with diabetes‐range HbA1c before admission and those detected during and after admission. In addition, this work adds to information on the most appropriate threshold to apply^8^ by stratifying maximum glucose levels in 1 mmol/L strata.

This retrospective cohort audit has several limitations. Firstly, the data are drawn from a single centre, although providing a comprehensive picture of hospital activity. Secondly, the interval between the random glucose test and subsequent HbA1c measurement varies. There may also be systematic bias existing within the population who received an HbA1c test following admission, for example guided by the presence of symptoms, treatments and other diseases. These variables could increase the likelihood of a person having diabetes and, therefore, the data could overestimate of the rate of diabetes in the non‐tested population.

Further limitations may arise from the suitability of HbA1c as a diagnostic test itself. Recent onset hyperglycaemia may not be associated with an immediate rise in HbA1c, potentially leading to false negatives, while haemoglobinopathies or a rapid erythrocyte turnover can also lead to an underestimation of glycation based on HbA1c.

### 4.4 Implications

HbA1c testing could be implemented for patients with high glucose levels (with electronic capture of measurements, real‐time algorithms to trigger HbA1c testing on existing or newly collected EDTA samples and clinician alerts) and managed within routine clinical care pathways including primary care. Such an approach has the potential to detect previously undiagnosed diabetes, prompt earlier diagnosis and treatment, and could help prevent or delay the onset of target organ damage.

For the UK National Health Service tertiary hospital trust (with just more than 1000 beds) in which this study was performed, HbA1c testing for all individuals with a random glucose ≥8 mmol/L would require 254 HbA1c tests being performed each week; if this threshold for testing were increased by 1 mmol/L to ≥9 mmol/L this would then require 188 HbA1c tests each week (Table 3).

This work was carried out retrospectively on routine laboratory data, and whilst we have reported HbA1c in the diabetes range, we do not have further information about whether these individuals have been diagnosed with diabetes. Further work on this will be possible in a future study using prospectively linked data. Translating these findings into a clinical strategy would require further work to predict risk of clinically important and actionable levels of persisting hyperglycaemia (including those at high risk of diabetes), potentially on the basis not only of glucose measurement, but other demographic and clinical information.

Implementing a system where HbA1c measurements were triggered by elevated glucose measurements would still require further assessment and tests before confirming a diagnosis of being at high risk or having diabetes. Challenges to implementation of testing triggered by an algorithm include resourcing a downstream clinical pathway that would include communication between secondary care and primary care settings, and communication with affected individuals about the diagnosis, establishing management plans, monitoring and follow‐up. There may be resource implications for such a testing strategy; the glucose threshold that triggers automated HbA1c testing should achieve an acceptable balance between test sensitivity and financial and logistical burden to laboratories and health services. The use of electronic health records allows for protocol‐led communication to primary care, which could potentially reduce the burden on hospital‐based clinicians in terms of managing the volume of investigations and communicating outcomes to the participant and GP (e.g. in ensuring results of routine blood tests are available).

## AUTHOR CONTRIBUTIONS

AF and BS conceived and designed the study. BS obtained and analysed the data. AF drafted the manuscript with LA. All authors contributed to interpretation of data and editing the manuscript. We are grateful for the comments of two anonymous reviewers that have improved the manuscript.

## FUNDING INFORMATION

AF and RR are supported by the National Institutes for Health and Care Research Oxford Biomedical Research Centre. LCA is funded by a Wellcome Trust Doctoral Research Fellowship [203921/Z/16/Z]. For the purpose of open access, the author has applied a CC BY public copyright license to any Author Accepted Manuscript version arising from this submission.

## CONFLICT OF INTEREST

None reported.

## References

[1] Levi OU , Webb F , Simmons D . Diabetes detection and communication among patients admitted through the emergency Department of a Public Hospital. Int J Environ Res Public Health. 2020;17(3):980. 10.3390/ijerph17030980 PMC7038107 32033242

[2] Forti P , Maioli F , Arnone G , et al. Age‐specific rate of undiagnosed diabetes and prediabetes in acute stroke. Diabetes Res Clin Pract. 2020;159:107968. 31830515 10.1016/j.diabres.2019.107968

[3] Sop J , Gustafson M , Rorrer C , Tager A , Annie FH . Undiagnosed diabetes in patients admitted to a clinical decision unit from the emergency department: a retrospective review. Cureus. 2018;10(10):e3390. 30533325 10.7759/cureus.3390 PMC6279010

[4] Manley SE , O'Brien KT , Quinlan D , et al. Can HbA1c detect undiagnosed diabetes in acute medical hospital admissions? Diabetes Res Clin Pract. 2016;115:106‐114. 27012459 10.1016/j.diabres.2016.01.023

[5] McAllister DA , Hughes KA , Lone N , et al. Stress hyperglycaemia in hospitalised patients and their 3‐year risk of diabetes: a Scottish retrospective cohort study. PLoS Med. 2014;11(8):e1001708. 25136809 10.1371/journal.pmed.1001708 PMC4138030

[6] Cheung NW , Campbell LV , Fulcher GR , et al. Routine glucose assessment in the emergency department for detecting unrecognised diabetes: a cluster randomised trial. Med J Aust. 2019;211(10):454‐459. 31680269 10.5694/mja2.50394

[7] Valentine NA , Alhawassi TM , Roberts GW , Vora PP , Stranks SN , Doogue MP . Detecting undiagnosed diabetes using glycated haemoglobin: an automated screening test in hospitalised patients. Med J Aust. 2011;194(4):160‐164. 21401454 10.5694/j.1326-5377.2011.tb02954.x

[8] Thornton‐Swan TD , Armitage LC , Curtis AM , Farmer AJ . Assessment of glycaemic status in adult hospital patients for the detection of undiagnosed diabetes mellitus: a systematic review. Diabet Med. 2022;39(4):e14777. 34951710 10.1111/dme.14777 PMC9302131

[9] Ghosh S , Manley SE , Nightingale PG , et al. Prevalence of admission plasma glucose in 'diabetes' or 'at risk' ranges in hospital emergencies with no prior diagnosis of diabetes by gender, age and ethnicity. Endocrinol Diabetes Metab. 2020;3(3):e00140. 32704561 10.1002/edm2.140 PMC7375073

[10] Dhatariya K , James J , Kong MF , Berrington R , Joint British Diabetes Society (JBDS) for Inpatient Care Group and guidelines writing group . Diabetes at the front door. A guideline for dealing with glucose related emergencies at the time of acute hospital admission from the Joint British Diabetes Society (JBDS) for Inpatient Care Group. Diabet Med. 2020;37(9):1578–1589. 32279343 10.1111/dme.14304

[11] R Core Team . A Language and Environment for Statistical Computing. R Foundation for Statistical Computing; 2020.

[12] Seneviratne Epa D , Ng E , Schneider HG , Nagalingam V , Bach LA , Sztal‐Mazer S . Prevalence of hyperglycaemia without previously recognised diabetes mellitus in the emergency department and subsequent management: a retrospective cross‐sectional study. Intern Med J. 2020;50(11):1397‐1403. 31841261 10.1111/imj.14720

[13] Wilson SE , Rosella LC , Lipscombe LL , Manuel DG . The effectiveness and efficiency of diabetes screening in Ontario, Canada: a population‐based cohort study. BMC Public Health. 2010;10(1):506. 20727141 10.1186/1471-2458-10-506 PMC2936425

[14] Greaves CJ , Stead JW , Hattersley AT , Ewings P , Brown P , Evans PH . A simple pragmatic system for detecting new cases of type 2 diabetes and impaired fasting glycaemia in primary care. Fam Pract. 2004;21(1):57‐62. 14760046 10.1093/fampra/cmh113

